# Supplementary material for: Integrated Proteomic and Metabolomic Analysis of Muscle Atrophy Induced by Hindlimb Unloading
Source: Biomolecules. 2024 Dec 26;15(1):14. doi: 10.3390/biom15010014 (PMC11764416; doi:10.3390/biom15010014)
Supplement: Supplementary file 1 [file biomolecules-15-00014-s001.zip › biomolecules-3366921-supplementary.pdf]

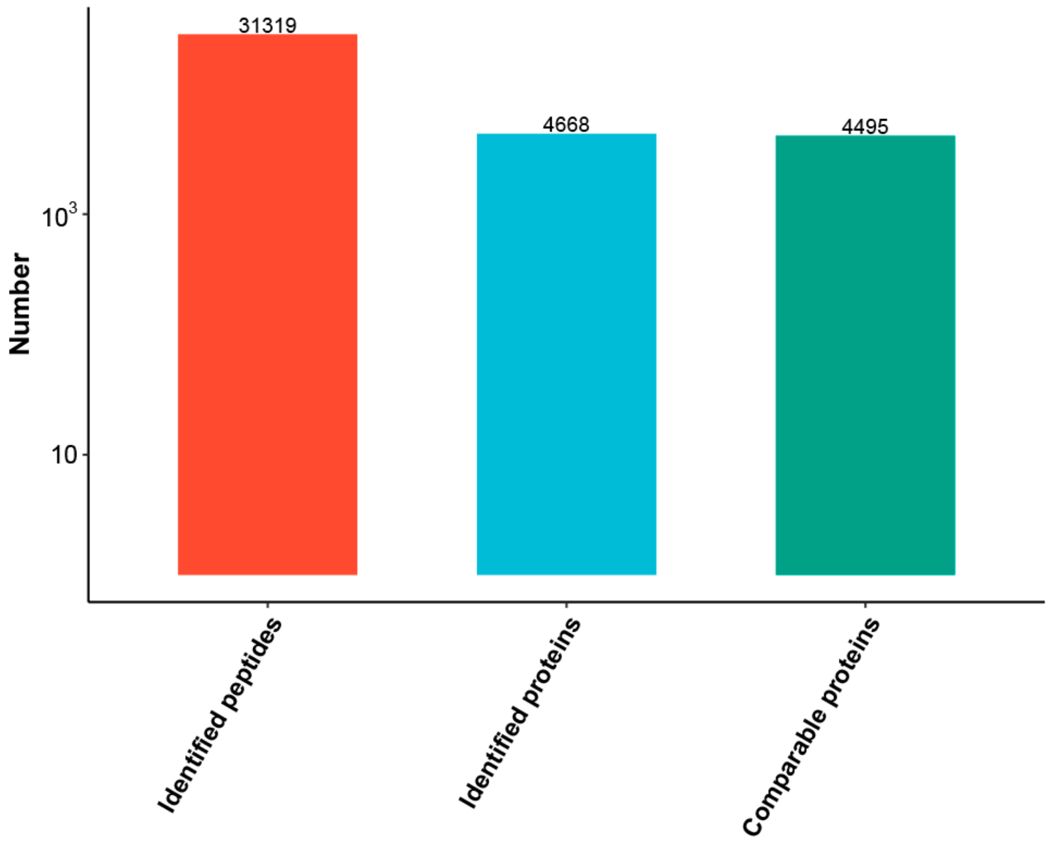

Figure S1. Peptide and protein identification overview. A total of 31319 peptides were identified, matching 4668 proteins with 4995 utilized for comparative analysis.

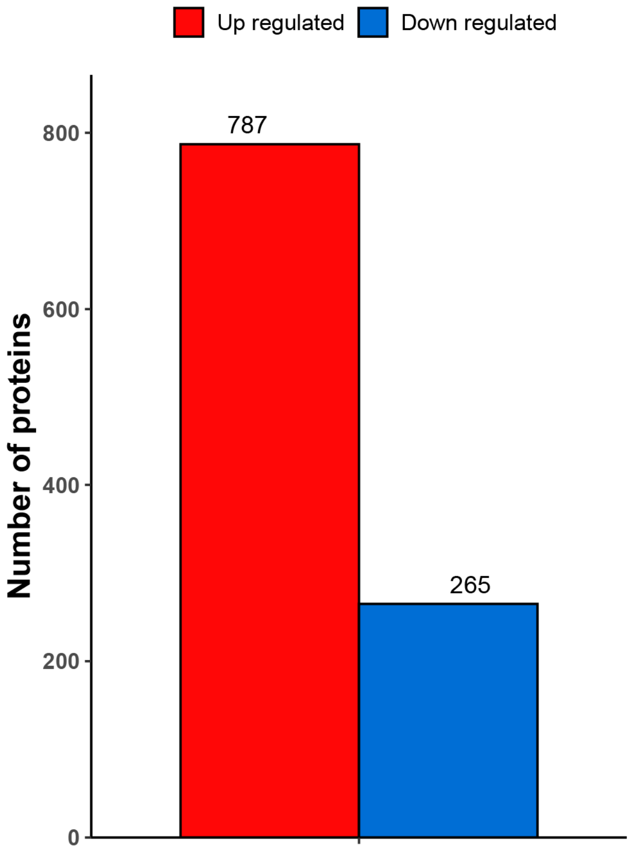

Figure S2. Comparative group differential information bar chart. Compared to the CON group, the HU group has 787 upregulated DEPs and 265 downregulated DEPs.

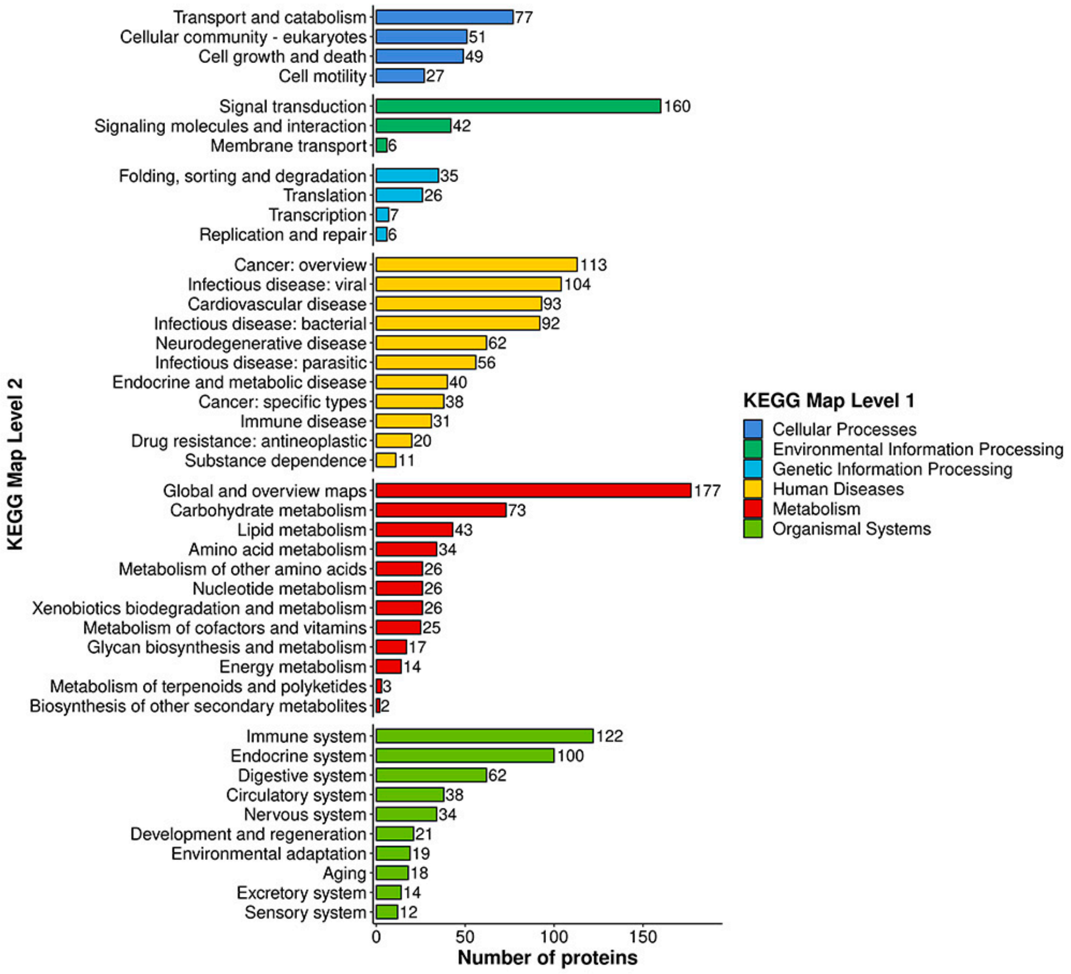

Figure S3. KEGG functional classification of DEPs.

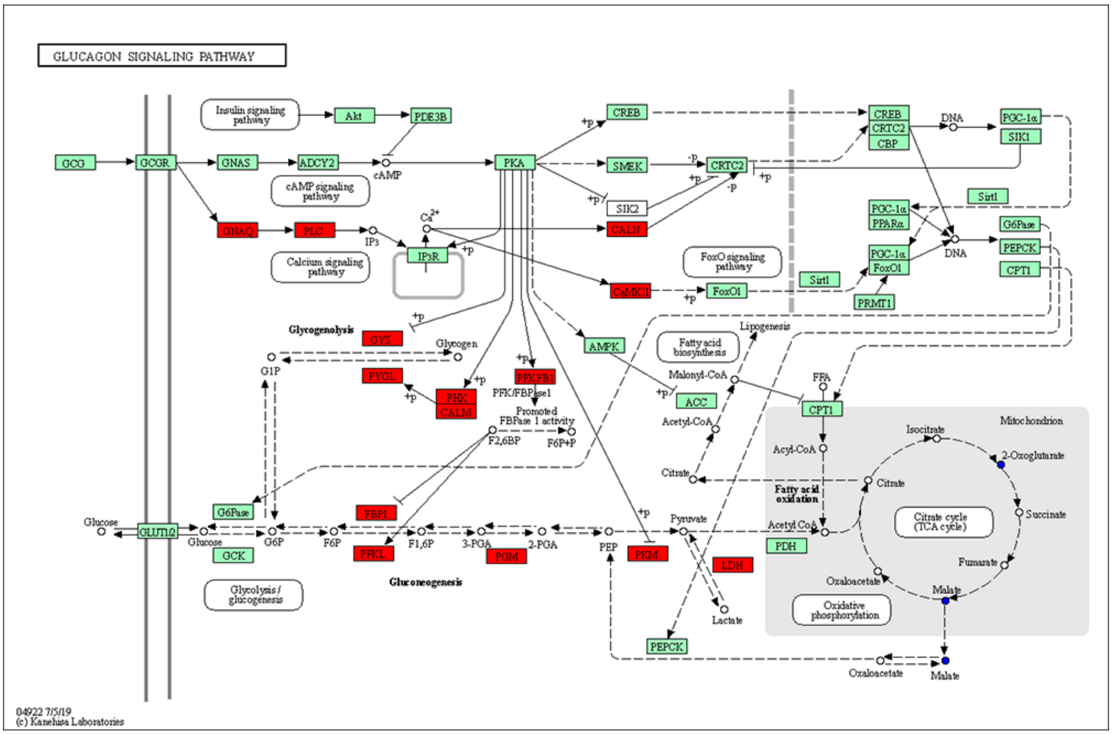

Figure S4. Glucagon signaling pathway map derived from the joint analysis of DEPs and DAMs.

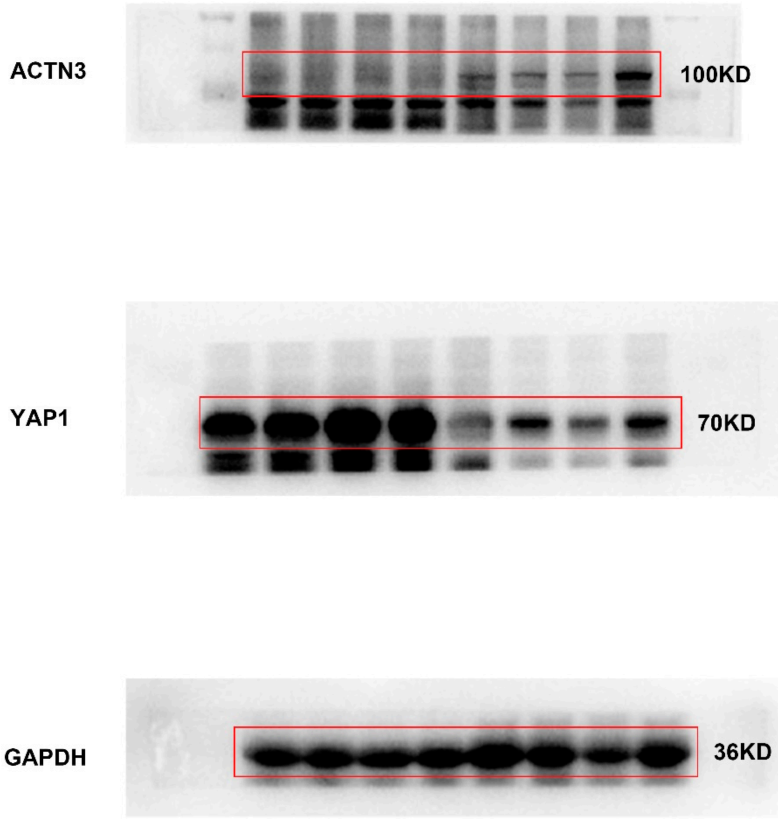

Figure S5. Western blot original images of Figure 3.

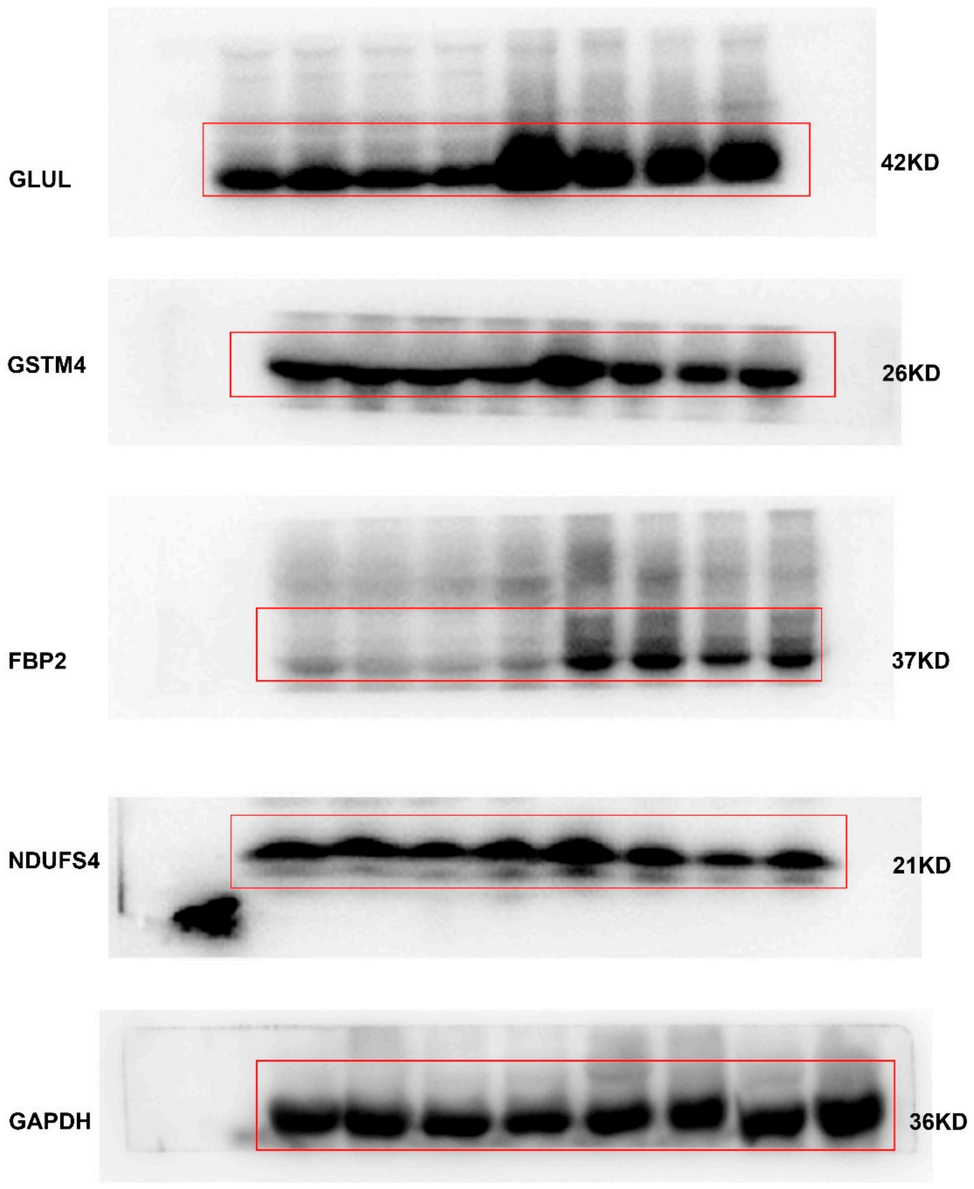

Figure S6. Western blot original images of Figue 4.
